# From Pre-Clinical Studies to Clinical Trials: Generation of Novel Therapies for Pregnancy Complications

**DOI:** 10.3390/ijms160612907

**Published:** 2015-06-08

**Authors:** Elizabeth C. Cottrell, Colin P. Sibley

**Affiliations:** 1 Maternal and Fetal Health Research Centre, Institute of Human Development, University of Manchester, Manchester M13 9WL, UK; E-Mail: colin.sibley@manchester.ac.uk; 2 Maternal and Fetal Health Research Centre, St. Mary’s Hospital, Central Manchester University Hospitals NHS Foundation Trust, Manchester Academic Health Science Centre, Manchester M13 9WL, UK

**Keywords:** pregnancy, fetal growth restriction, preeclampsia, preclinical trials in pregnancy

## Abstract

Complications of pregnancy represent a significant disease burden, with both immediate and lasting consequences for mother and baby. Two key pregnancy complications, fetal growth restriction (FGR) and preeclampsia (PE), together affect around 10%–15% of all pregnancies worldwide. Despite this high incidence, there are currently no therapies available to treat these pregnancy disorders. Early delivery remains the only intervention to reduce the risk of severe maternal complications and/or stillbirth of the baby; however early delivery itself is associated with increased risk of neonatal mortality and morbidity. As such, there is a pressing need to develop new and effective treatments that can prevent or treat FGR and PE. Animal models have been essential in identifying and screening potential new therapies in this field. In this review, we address recent progress that has been made in developing therapeutic strategies for pregnancy disorders, some of which are now entering clinical trials.

## 1. Introduction

Achieving an optimal pregnancy outcome–the birth of a healthy, appropriately grown baby at term–is the primary goal in any pregnancy. Fetal growth restriction (FGR), the failure of a fetus to achieve its genetic growth potential, affects between 5% and 10% of pregnancies [1]. In addition to a ~4-fold increased risk of stillbirth [2], babies born small are at a significantly greater risk of developing disabilities in childhood (such as cerebral palsy; [3]) and diseases in later life (including high blood pressure or diabetes; [4]). Preeclampsia (PE), defined by the onset of hypertension and proteinuria after 20 weeks of gestation, occurs in around 3%–5% of pregnancies, and significantly increases the risk of pre-term delivery and FGR, as well as endangering maternal survival [5]. The societal and economic impacts of these pregnancy complications are therefore hugely significant, and the development of treatment strategies to prevent or reduce disease progression remains a priority. In recent years, marked progress has been made in addressing this aim, as we better understand the underlying mechanisms that contribute to these pregnancy disorders. The use of animal models has been pivotal, both in advancing our understanding of normal pregnancy physiology and pathophysiology, as well as in the testing of new treatment strategies. Assessment of pregnancy outcomes following specific treatments or interventions in relevant animal models has provided critical preclinical efficacy and safety information prior to translation to clinical trials. This review aims to provide an overview of recent advances that have been made in this field using a variety of animal models of FGR and PE, including the translation of several of these findings to clinical trials that are now underway.

## 2. Physiology of Normal Pregnancy and Pathophysiology in Pregnancy Disorders

It is clear that genetic as well as environmental factors are both important determinants of pregnancy outcome. A viable pregnancy is dependent on both the fetal genotype, as well as an appropriate maternal adaptation to pregnancy and sustained maternal health throughout gestation. Of key importance, successful pregnancy is critically dependent on adequate remodelling of the maternal cardiovascular system. During normal pregnancy, a reduction in peripheral vascular resistance is accompanied by expansion of maternal blood volume (~30%–50%) and increased cardiac output. Uterine artery remodelling in early pregnancy allows redistribution of maternal cardiac output to favour utero-placental blood flow and hence maximise oxygen and nutrient delivery to the fetus [6]. In terms of pregnancy pathology, both FGR and PE are often associated with impaired uteroplacental blood flow and vascular dysfunction. The underlying aetiologies are complex, but multiple lines of evidence indicate that reduced trophoblast invasion in early pregnancy and poor placentation might represent a common mechanism [7]. As a consequence, placental development and function is impaired, leading to raised utero- and fetoplacental vascular resistance, irregular placental blood flow (which can itself trigger ischaemic-reperfusion injury within placental tissue and overproduction of reactive oxygen species; ROS) and reduced capacity for placental exchange of nutrients and fetal products of metabolism. Additionally, in a number of FGR cases, alterations in placental morphology and/or nutrient exchange capacity may underlie the pathology, in the absence of blood flow defects [8]. Regardless of the underlying pathology, the resultant placental dysfunction is a causal factor for impaired fetal growth. Consequently, identifying treatments that improve uteroplacental perfusion and/or placental exchange capacity remains a central research focus for improving fetal growth in pregnancies complicated by FGR and PE.

## 3. Use of Animal Models to Study Pregnancy Disorders

Whilst certain aspects of pregnancy research are amenable to *in vitro* or *ex vivo* approaches, cellular models are not able to recapitulate the complexity of pregnancy, where maternal, placental and fetal physiologies interact. An intact, mammalian system is therefore essential to study uteroplacental function and fetal development *in vivo* and is critical for the screening of potential therapies for use in pregnancy*.* Animal models have thus been used extensively to study the processes underlying both normal physiological adaptations to pregnancy, as well as to increase our understanding of key genetic and environmental contributors to the development of pregnancy disease. A range of animal models has been used to study these processes. Some of the more commonly used large animal models include ovine and swine. Advantages of using these larger animals include their tempo of development, which is similar to that of the human fetus, and the potential to perform surgeries enabling access to both maternal and fetal tissues, for measurement of specific factors in the blood (not always possible to measure with small volumes) or for delivery of agents directly into either circulation. However, disadvantages to using these models include the relatively long gestation, high maintenance and infrastructure costs, as well as a placental structure and morphology that is significantly different from that of the human placenta. Non-human primates (e.g., macaques) have also been used in some studies. Although these are clearly more closely related to humans, significant ethical considerations arise, in addition to the need for specialist facilities and associated costs.

Small animal models, such as guinea pigs, rats and mice have been used more extensively, with advantages in terms of short gestation and reduced housing and maintenance costs. In terms of placental morphology, the guinea pig most closely resembles that of the human. However, the many similarities in terms of placental structure and function between humans and mice (reviewed in detail elsewhere; see [9]), coupled with the ease of genetic manipulation in this species, has significantly improved our understanding of disorders of pregnancy, including FGR and PE. Limitations of using mice as a model in pregnancy research include their multiparous nature and markedly different developmental trajectories in terms of pre- and postnatal organ development compared with humans, as well as some differences in placental structure (e.g., mouse placenta is trichorial whereas human is monochorial). Despite these differences, use of mouse models in pregnancy research has enabled significant progress to be achieved in the development of new treatment strategies, discussed in more detail below.

## 4. Experimental Approaches to Induce FGR

An excellent and comprehensive review of animal models used to study FGR, as well as their limitations, has recently been published (see [10]). To briefly summarise, dietary restriction, exposure to hypoxia, pharmacological manipulations and surgical interventions are all commonly used to generate animal models of FGR. Maternal undernutrition–achieved either through total caloric or protein restriction–induces FGR in a range of species (sheep, guinea pigs, rats and mice, as well as in non-human primates), with the degree of severity being proportional to the degree of restriction, but with species and gestational stage-dependent effects. Exposure of pregnant animals to hypoxia during gestation can also impair fetal growth, and this approach has been used widely in ovine and rat models, as well as in the chick embryo, where direct effects of hypoxia on the fetus can be determined independent of effects on maternal physiology. As for the effects of nutrition, the variability in terms of degree of FGR achieved is specific to the animal model used, the severity of hypoxia and the length of exposure. In terms of pharmacological manipulations, administration to the mother of glucocorticoids or the nitric oxide synthase (NOS) enzyme inhibitor, nitro-l-Arg-methyl ester (l-NAME), are two of the most commonly used interventions that robustly induce FGR. Of note, there is likely significant overlap between the dietary and pharmacological approaches described above in terms of the mechanisms by which FGR is induced. Frequently, manipulations that induce FGR do so, at least in part, through a reduction in uteroplacental blood flow. As mentioned previously, this reduction in uteroplacental perfusion is also a common feature of human FGR. As such, surgical models that have been developed to induce FGR do so by directly disrupting uterine blood flow, either through ligation or occlusion of the uterine artery. Together these approaches provide a range of FGR models that can be interrogated both in terms of determining the role of specific pathways that contribute to the observed growth restriction, as well as identifying potential treatment strategies.

## 5. Genetic Mouse Models of FGR

In addition to the intervention models outlined above, genetic models have been extremely valuable in determining the importance of specific genes and/or pathways in the development of FGR. There are now several well-characterised genetic mouse models that provide a reproducible degree of FGR, and with pathophysiological features similar to the human disease, removing the need for maternal intervention. Broadly speaking, the majority of genetic models that have been widely used as models of FGR are associated either with altered angiogenic/vascular function, or impaired growth factor signalling [9]. The latter are generally associated with alterations in key fetal growth regulators such as insulin-like growth factor 1 and 2 (IGF-1, IGF-2). Although beyond the scope of this review, imprinted genes play a key role in the regulation of fetoplacental growth, largely via alterations in fetal and/or placental IGF signalling. For example, recent evidence has shown that disruption of a key imprinting cluster on mouse chromosome 12 leads to significant FGR, associated with impaired IGF signalling at multiple levels [11].

In our laboratory, we have made extensive use of two genetic mouse models of FGR. The first of these, the placental-specific insulin-like growth factor 2 knockout (IGF2-P0) mouse, lacks the placental-specific transcript of IGF2, an imprinted gene playing a major role in the promotion of fetal and placental growth. This deletion leads to significant and early impairment in placental growth and morphology, from embryonic day 12; E12 [12]. In the fetus, growth restriction is apparent from E16, and at term affected littermates are approximately 30% lighter than their wildtype (WT) counterparts. In this model, defects in placental nutrient exchange capacity are thought to be the predominant cause of FGR. The second FGR model used extensively by us, and others, is the endothelial nitric oxide synthase knockout (eNOS^−/−^) mouse. Here, deletion of eNOS significantly impairs the pregnancy-induced alterations in maternal cardiovascular function [13], and both uterine and umbilical artery blood flows are reduced [14,15]. FGR is apparent from E17, with a 15%–20% reduction in fetal weight compared with the background strain, C57BL/6J WT controls [15,16,17]. In addition, impaired system A amino acid transport is seen in placentas of eNOS^−/−^ animals [16]. Together, these mouse models enable us to test the efficacy of potential therapies that may improve blood flow and/or placental exchange capacity in FGR (discussed in more detail below).

## 6. Experimental Models of PE

Despite the fact that PE is widely considered a human-specific pregnancy disorder, numerous animal models have been generated with the aim of mimicking this disease. These animal models have been generated based on current knowledge regarding causes of, or mechanisms involved in, PE development. Ideally, an appropriate model of PE should demonstrate pregnancy-specific maternal hypertension, endothelial dysfunction, perturbation of factors key to angiogenic balance and proteinuria [18].

Similar to the FGR models discussed above, a variety of maternal interventions have been used in an attempt to reproduce key aspects of PE [18]. These include uteroplacental blood flow disruption (e.g., the Reduced Uterine Perfusion Pressure; RUPP model), pharmacological manipulations (e.g., inhibition of eNOS) and infusion of anti-angiogenic factors. Overall, these interventions are used largely to induce the vascular dysfunction and reduction in placental perfusion that are considered key components of PE pathology. The RUPP model consistently produces a reduction in uteroplacental perfusion, a pregnancy-specific increase in maternal blood pressure and reproduces the angiogenic factor imbalances seen in human PE (*i.e.*, an increase in the release of soluble fms-like tyrosine kinase 1 (sFlt-1) and reduced vascular endothelial growth factor (VEGF) and placental growth factor (PlGF; [19]). Global inhibition of NOS, using chronic infusion of l-NAME, also leads to a PE-like phenotype, with increased maternal blood pressure, proteinuria and impaired kidney function [20,21,22]. More recently, several new models have been developed in which sFlt-1 is either directly infused [23,24], adenovirally delivered [25] or transgenically over-expressed [26], to demonstrate the key role of this angiogenic imbalance in PE-like phenotype. The latter of these models also leads to a reduction in fetal growth, again recapitulating an important endpoint seen in most human PE cases.

There are now also a considerable number of genetic mouse models that have been observed or engineered to have PE-like phenotypes. Although not an exhaustive list, these include the *Corin* knockout mouse, which exhibits reduced trophoblast invasion, maternal hypertension and proteinuria [27]; the catechol-*O*-methyltransferase knockout mouse (COMT^−/−^), which has elevated maternal blood pressure, impaired kidney morphology, proteinuria, and elevated sFlt-1 [28], and the STOX-1 transgenic mouse, in which mice are engineered to over-express the human pre-eclampsia-associated gene, STOX-1, and strongly recapitulate the human disease [29]. A final interesting model is the inbred BPH/5 mouse, in which females have mildly elevated blood pressure throughout adult life, but during gestation this rises markedly, accompanied by the onset of proteinuria [30]. Both fetal and placental weights are reduced in this model, associated with impairments in placental vascular development [31].

Although these animal models have been highly informative in terms of progressing our understanding of components of PE, very few adequately reproduce the impaired trophoblast invasion characteristic of the human disease [18]. However, preclinical studies carried out in these models have provided encouraging data on potential therapeutics for PE. As our understanding of this disease improves, and a greater number of the potential genetic loci involved are identified, it is envisaged that this will lead to the development of more targeted, specific genetic models that hopefully mimic more closely the human disease.

## 7. Approaches Used to Develop Treatments for FGR and PE

To date, the pharmaceutical industry has not invested in developing treatments for pregnancy conditions, at least in part due to potential risk of fetal toxicity. However, there are drugs developed for non-pregnancy conditions (such as cardiovascular disease), which have modes of action potentially useful in ameliorating the pathologies implicated in FGR and PE. We and others have tested a number of such drugs, using a range of different animal models, including those outlined above. Our strategy has been to focus initially on treatments that are currently either in clinical use or in clinical trials in non-pregnant individuals, given that these are most likely to have the greatest potential for translation to clinical trials in pregnant women. Below, we discuss a range of potential treatments that have been used with the primary intention of either improving fetal and/or placental weight or preventing maternal symptoms of PE (*i.e.*, high blood pressure, proteinuria, excessive production of ROS).

## 8. Treatment Strategies Enhancing Nitric Oxide Bioavailability or Action

It is clear from both clinical and experimental data that the impairment of uteroplacental blood flow is a key factor in both FGR and PE. A wealth of evidence indicates that nitric oxide (NO) signalling is critical for maintenance of the low resistance/high flow uteroplacental system that favours fetal oxygen and nutrient delivery. NO is the key vasodilator in both the maternal resistance vessels controlling blood flow to the placenta [32], as well as in the fetoplacental resistance vessels [33] and there is considerable evidence for reduced production and/or bioavailability of NO in both FGR and PE [34,35]. Given this evidence, it is not surprising that there has been a considerable focus on the development of interventions that either increase NO production and/or enhance downstream NO signalling pathways.

Endogenously, NO is derived from oxidation of the amino acid l*-*arginine by the NOS enzymes, of which there are three isoforms (neuronal, inducible and endothelial; nNOS, iNOS and eNOS, respectively). Several groups have shown that maternal supplementation with l*-*arginine, by increasing the NOS substrate availability, is able to improve fetal weight in models of maternal nutrient restriction [36] and hypoxia-induced FGR [37]. However, this has not been a consistent finding amongst different studies [38], likely reflecting differences in efficacy between different models and treatment regimes. In terms of PE models, maternal l*-*arginine supplementation was found to partially reverse the hypertension and completely prevent the increase in renal endothelin-1 mRNA levels associated with sFlt-1 infusion [23]. There have been a small number of clinical trials investigating the utility of maternal l*-*arginine supplementation in both FGR and PE; these have shown some promise, with a reduction in maternal hypertension, improved vascular function and increases in fetal weight demonstrated [39,40,41,42].

In terms of direct NO-donor drug administration (e.g., glyceryl trinitrate), there have been some beneficial effects seen in a few clinical studies, mainly in terms of improved maternal blood pressure regulation and uterine blood flow in at-risk pregnancies (see [43] for review). However, these interventions did not result in a reduced incidence of PE development or significant improvements in fetal growth. In addition, significant side-effects may preclude the usefulness of NO donor drugs in large-scale studies (e.g., headache and/or hypotension), due to issues of maternal compliance.

Perhaps the most promising potential therapy to date, in terms of increasing NO actions, is sildenafil citrate (SC). Originally developed to treat pulmonary hypertension, SC acts by inhibiting the phosphodiesterase (PDE) type 5 enzyme which catalyses the breakdown of cyclic GMP, prolonging the actions of NO throughout the body and enhancing NO-dependent vasodilation. Given these mechanisms of action, numerous studies have investigated the potential for SC to improve fetal growth and pregnancy outcome, with some significant beneficial effects demonstrated in a range of models [44]. In isolated myometrial arteries from FGR pregnancies, incubation with SC significantly improved vascular function, reducing constriction and enhancing vasodilation [45]. In the COMT^−/−^ mouse model, SC administration during the final third of pregnancy rescued fetal growth, likely through a restoration of umbilical blood flow [46]. We have also recently demonstrated that drinking water administration of SC in the IGF2-P0 mouse model of FGR significantly improved fetal weight, specifically in the growth-restricted littermates; this was associated with improved amino acid clearance across a larger placenta [47]. In two l-NAME-induced PE-like models, SC treatment significantly reduced the levels of sFlt-1 [48] and improved fetal growth [49] compared with untreated PE groups.

In terms of clinical studies, cases in which SC was used in pregnant women for the treatment of pulmonary hypertension provided important safety data regarding the use of this drug in pregnancy [50,51]. To date, there have been several small-scale clinical trials indicating beneficial effects of maternal oral SC treatment on fetal growth [52,53]. In light of positive data from both preclinical experimental studies and these small clinical trials, the STRIDER study (Sildenafil Therapy In Dismal prognosis Early-onset intrauterine growth Restriction) was launched in 2014 [54]. STRIDER is the first large-scale randomised controlled trial (RCT) that aims to investigate the effectiveness of SC therapy to improve pregnancy outcomes in severe early-onset FGR pregnancies (primary outcome: birth of a live infant, without adverse neonatal outcome). This study involves trials in a number of countries, and outcome data is expected by 2020.

## 9. Significant Promise of Antioxidant Therapies in FGR and PE

In addition to agents targeting the NO pathway directly, the other broad class of potential treatments that have shown promise in preclinical models of pregnancy complications are those that enhance antioxidant defenses. Increased oxidative damage in the placenta is a major feature of both FGR and PE [55], and is likely causal in both impairing vascular function as well as nutrient transport.

A range of different antioxidant agents have been used to date in pregnancy research, with the aim of reducing ROS production or damage in FGR and PE. These include mimetics of free radical scavengers, such as tempol, a superoxide dismutase (SOD) mimetic. In both the BPH/5 [56] and eNOS^−/−^ [17] mouse models, maternal tempol administration improved maternal blood pressure regulation/blood flow defects and increased fetal weight. It was also associated with normalization of ROS levels in the placenta in BPH/5 animals [56]. The efficacy of resveratrol, a plant polyphenol that has been shown to have numerous beneficial effects on cardiovascular function at least in part through reducing oxidative stress, has also been assessed in animal models of pregnancy disorders. Results have been conflicting, and are likely dependent on the model used and parameter assessed. One study demonstrated that maternal resveratrol supplementation was able to rescue the blood flow defects in COMT^−/−^ but not eNOS^−/−^ pregnancies, however fetal growth was improved overall in both strains [57]. Of concern, resveratrol supplementation in a non-human primate model was found to markedly alter fetal pancreatic morphology, the long-term consequences of which are currently unknown [58]. Thus, although some likely beneficial effects are seen with maternal resveratrol supplementation, caution must be exercised and at present this treatment is certainly not suitable for use in pregnant women.

There has also been significant interest in the potential for antioxidant vitamin supplementation (notably vitamins C and E) in preventing and/or treating PE or impaired fetal growth. There is some evidence that supplementation with vitamin C can reduce the blood flow defects associated with NOS inhibition in sheep [59]. There is also some evidence that the long-term detrimental effects of prenatal glucocorticoid treatment might be reversed by the administration of vitamins C and E during pregnancy in the rat [60], however in this study, some detrimental effects of vitamin supplementation were seen in control animals.

In terms of translation to humans, a number of studies have assessed the efficacy of vitamin C and/or E supplementation in women to reduce the incidence or severity of PE. These studies have found conflicting results. Beneficial effects of vitamins C and E supplementation were seen in a trial of women at high-risk of PE, with a reduction in incidence of PE development in those receiving supplementation [61] and improvements in terms of the biochemical profile of the disease [62]. However, a larger study performed with an equivalent supplementation strategy did not identify any reduction in incidence in the treated group compared with placebo, and in fact vitamin supplementation was associated with a reduction in fetal weight [63]. As such, supplementation with vitamins C and E in clinical trials as a means of preventing PE development has now been abandoned.

Melatonin, a neurohormone produced primarily by the pineal gland, has marked antioxidant properties in addition to its key role in the regulation of seasonal and circadian cycles. Melatonin can act both as a free radical scavenger as well as having beneficial indirect effects, through upregulating cellular antioxidant enzyme systems, including glutathione-reductase, superoxide dismutase and catalase [64]. In animal models, maternal melatonin administration has been shown to reduce the adverse effects of perinatal hypoxia in sheep [65], and to improve fetal growth and placental antioxidant defense enzyme expression in a rat model of FGR induced by maternal undernutrition [66].

Similar to the case of SC, above, the significant preclinical evidence for beneficial effects of melatonin to improve fetal outcomes in complicated pregnancies has recently prompted the initiation of two clinical trials. The first of these aims to assess whether melatonin treatment can safely and effectively prolong pregnancy in women with preterm PE [67]. The second trial aims to assess whether melatonin treatment in women with severe early-onset FGR can reduce levels of oxidative stress in both maternal and fetal circulation, as well as within the placenta [68]. Both of these trials are Phase I pilot studies; if data are consistent with protective and beneficial effects of melatonin treatment in pregnancy, these findings will likely underpin future large-scale RCTs.

## 10. Other Beneficial Interventions in Preclinical Models of FGR and PE

Additional treatments that have shown promise as potential therapies for FGR and/or PE include low-dose aspirin, statins and dietary nitrate supplementation. Furthermore, a growing body of literature is emerging demonstrating the importance of maternal vitamin D levels in human pregnancy. There is some evidence to suggest that maternal vitamin D deficiency can increase the risk of PE [69] and FGR [70], and that supplementation may be beneficial in reducing the risk of these pregnancy complications [71]. However, the current evidence remains inconclusive [71], and future preclinical studies and well-controlled intervention trials are needed.

In terms of aspirin administration, this has been used largely in order to reduce the risk of gestational hypertension/PE development, through effects that are presumed to alter the haemodynamic properties of blood (*i.e.*, anticoagulant and antiplatelet effects). In the STOX-1 transgenic mouse model of PE, maternal low-dose aspirin treatment from the beginning of pregnancy was found to be highly effective in ameliorating the elevated maternal blood pressure and proteinuria in this model [29]. Although there remains relatively limited clinical evidence, a recent meta-analysis indicated there is a significant effect of low-dose aspirin treatment (75–100 mg/day) to reduce the risk of PE development in pregnant women [72].

Ongoing research into the use of statin therapy for the prevention of PE is also showing some promise. Statins are a class of drugs developed to inhibit endogenous cholesterol synthesis, and are currently widely prescribed for the prevention or treatment of cardiovascular disease. In addition to these effects, there is also evidence for beneficial actions of statins to increase antioxidant defense, improve vascular function and reduce platelet aggregation [73]. In several animal models of PE, including the RUPP rat model and mouse models of sFlt-1 over-expression, maternal treatment with pravastatin significantly reduced sFlt-1 levels, increased PlGF, improved vascular function, reduced oxidative stress, attenuated maternal hypertension and renal dysfunction and improved fetal growth [26,74,75,76]. However, detrimental effects of statins on placental function have also been reported [77,78], and as such there remains controversy regarding the utility of statin therapy in pregnancy. Currently ongoing is the first clinical trial assessing the effects of statin therapy to improve pregnancy outcomes in PE (the StAmP trial; Statins to Ameliorate early-onset Preeclampsia; ISRCTN23410175); the results of this will go some way to determining whether this approach may be of benefit in women.

Dietary nitrate supplementation, providing nitrite, a source of NO-generating potential, represents another promising potential therapy for use in pregnancy. Dietary nitrate has to date been used in a number of preclinical and clinical studies in non-pregnant individuals and shown to improve blood pressure regulation, increase blood flow, enhance exercise performance and protect against damage caused by ischemia-reperfusion injury [79]. We have recently shown that maternal dietary nitrate supplementation, via beetroot juice, improves maternal vascular function and fetal growth in the eNOS^−/−^ mouse [80,81]. In addition to this promising data so far, dietary constituents more generally represent an appealing avenue for treatment of pregnancy-related disease, being easy to administer and having the potential for improved maternal compliance compared with pharmacological interventions, which is a significant problem in pregnant women [82].

## 11. Future Strategies for Development of Pregnancy Treatments: Targeted Approaches

In addition to systemic therapeutic approaches discussed above (and summarised in Table 1, below), significant progress has also been made in recent years to develop targeted treatments that improve uteroplacental blood flow and/or enhance placental efficiency. These aim to directly target drugs or therapies to specific sites, improving efficacy and potentially reducing adverse effects of systemically delivered agents. One approach that has been used successfully to date has employed an adenoviral-mediated gene delivery system, to locally overexpress the angiogenic and vasodilatory agent VEGF within the maternal uterine vasculature. Delivery of adenoviral-VEGF (Ad-VEGF) at mid-gestation to the pregnant sheep increased uterine artery (UtA) blood flow, improved *ex vivo* UtA vascular function and increased NOS expression [83,84]. Furthermore, using this same delivery system in an ovine model of FGR, it has recently been shown that improvements in UtA vascular function following Ad-VEGF therapy associated with increased fetal growth velocity and a reduction in the number of growth restricted fetuses [85]. These preclinical findings underpin the recently commenced EVERREST trial (see https://clinicaltrials.gov/ct2/show/NCT02097667), which over the next five years aims to test the efficacy of Ad-VEGF gene therapy to improve fetal growth in pregnancies affected by severe early onset FGR.

**Table 1:** Summary of treatment strategies used in preclinical models of fetal growth restriction (FGR) and preeclampsia (PE), and their translation to clinical trials (where applicable).

Treatment	Model	Effect (Reference)
l-arginine	***Preclinical studies***	
	Maternal nutrient restriction (FGR)	Increased fetal weight [36].
	Maternal hypoxia (FGR)	Increased fetal weight [37].
	sFlt-1 infused rat (PE)	Reduced blood pressure [23].
	***Clinical trials***	
	PE	Reduced blood pressure [39,42].
	FGR	Increased fetal weight [40,41].
Sildenafil	***Preclinical studies***	
	COMT^−/−^ mouse (PE)	Improved blood flow, increased fetal weight [46].
	Igf2-P0 mouse (FGR)	Increased fetal weight [47].
	l-NAME infused rat (PE)	Increased fetal weight [49].
	***Clinical Trials***	
	PE	Reduction in maternal blood pressures; non-significant increase in fetal weight [52].
	Early-onset FGR	Increased abdominal circumference [53].
	Severe early-onset FGR	Ongoing RCT, *STRIDER* [54].
Tempol	***Preclinical studies***	
	eNOS^−/−^ mouse (FGR)	Improved blood flow, increased fetal weight [17].
	BPH/5 mouse (PE)	Prevention of maternal hypertension and proteinuria, increased fetal weight [56].
	***Clinical Trials***	
	No known clinical trials in pregnancy	
Resveratrol	***Preclinical studies***	
	eNOS^−/−^ mouse (FGR)	Increased fetal weight [57].
	COMT^−/−^ mouse (PE)	Improved blood flow, increased fetal weight [57].
	***Clinical Trials***	
	No known clinical trials in pregnancy; some evidence of potential adverse effects in non-human primate [58]	
Melatonin	***Preclinical studies***	
	Maternal hypoxia (FGR)	Reduced oxidative damage and improved neurodevelopment [65].
	Maternal nutrient restriction (FGR)	Increased birth weight [66].
	***Clinical Trials***	
	PE	Ongoing RCT [67].
	Early-onset FGR	Ongoing RCT [68].
Aspirin	***Preclinical studies***	
	STOX-1 transgenic mouse (PE)	Reduced blood pressure/proteinuria [29].
	***Clinical Trials***	
	Women at risk of PE	Systematic review of evidence to date indicates reduced risk of PE in women taking aspirin [72].
Statins	***Preclinical studies***	
	RUPP model (PE)	Reduced maternal blood pressure, increased fetal weight [76].
	sFlt-1 over-expressing mouse (PE)	Reduced maternal blood pressure, proteinuria and increased fetal weight [26].
	***Clinical Trials***	
	Severe PE	Ongoing RCT, StAmP trial; ISRCTN23410175.

An alternative approach, also currently under development, is exploiting the use of homing peptide sequences to achieve targeted drug delivery. Specific peptide sequences that bind selectively to the placenta and uterine vasculature have recently been identified [86], and used to deliver targeted nanoparticles to human placental tissue *in vitro*, as well as to the mouse placenta *in vivo* [87,88]*.* These findings provide a promising avenue for future therapeutic approaches in pregnant women, with the potential for improved drug efficacy, reduced risk of fetotoxicity and reduced maternal side effects compared with systemic administration.

## 12. Conclusions and Future Avenues for Research

The use of animal models of FGR and PE to test potential therapeutics has to date been largely successful. Several clinical trials are now underway as a direct result of the promising data generated from these preclinical studies. Continuing to advance our understanding of factors that promote pregnancy disorders will provide an increasing range of potential avenues for intervention. In addition to finding treatment strategies to improve outcomes in pregnancies complicated by FGR and PE, there is an increasing need to address disorders of pregnancy associated with fetal overgrowth. In particular, excessive fetal growth is associated with maternal obesity and diabetes, both of which are a growing problem worldwide. Again, animal models will be essential in order to develop appropriate interventions or treatments in these pregnancies. A focus on improving maternal health before conception as well as during pregnancy, in addition to developing new treatment strategies for complicated pregnancies, will be key in order to improve both immediate and long-term offspring outcomes.

## References

[1] Bamfo J.E., Odibo A.O. (2011). Diagnosis and management of fetal growth restriction. J. Pregnancy.

[2] Gardosi J., Madurasinghe V., Williams M., Malik A., Francis A. (2013). Maternal and fetal risk factors for stillbirth: Population based study. BMJ.

[3] McIntire D.D., Bloom S.L., Casey B.M., Leveno K.J. (1999). Birth weight in relation to morbidity and mortality among newborn infants. N. Engl. J. Med..

[4] Cottrell E.C., Ozanne S.E. (2008). Early life programming of obesity and metabolic disease. Physiol. Behav..

[5] Sibai B., Dekker G., Kupferminc M. (2005). Pre-eclampsia. Lancet.

[6] Osol G., Mandala M. (2009). Maternal uterine vascular remodeling during pregnancy. Physiology.

[7] Chaddha V., Viero S., Huppertz B., Kingdom J. (2004). Developmental biology of the placenta and the origins of placental insufficiency. Semin. Fetal Neonatal Med..

[8] Sibley C.P., Turner M.A., Cetin I., Ayuk P., Boyd C.A., D’Souza S.W., Glazier J.D., Greenwood S.L., Jansson T., Powell T. (2005). Placental phenotypes of intrauterine growth. Pediatr. Res..

[9] Dilworth M.R., Sibley C.P. (2013). Review: Transport across the placenta of mice and women. Placenta.

[10] Swanson A.M., David A.L. (2015). Animal models of fetal growth restriction: Considerations for translational medicine. Placenta.

[11] Charalambous M., da Rocha S.T., Hernandez A., Ferguson-Smith A.C. (2014). Perturbations to the IGF1 growth pathway and adult energy homeostasis following disruption of mouse chromosome 12 imprinting. Acta Physiol..

[12] Constancia M., Hemberger M., Hughes J., Dean W., Ferguson-Smith A., Fundele R., Stewart F., Kelsey G., Fowden A., Sibley C. (2002). Placental-specific IGF-II is a major modulator of placental and fetal growth. Nature.

[13] Kulandavelu S., Qu D., Adamson S.L. (2006). Cardiovascular function in mice during normal pregnancy and in the absence of endothelial no synthase. Hypertension.

[14] Kulandavelu S., Whiteley K.J., Bainbridge S.A., Qu D., Adamson S.L. (2013). Endothelial no synthase augments fetoplacental blood flow, placental vascularization, and fetal growth in mice. Hypertension.

[15] Kulandavelu S., Whiteley K.J., Qu D., Mu J., Bainbridge S.A., Adamson S.L. (2012). Endothelial nitric oxide synthase deficiency reduces uterine blood flow, spiral artery elongation, and placental oxygenation in pregnant mice. Hypertension.

[16] Kusinski L.C., Stanley J.L., Dilworth M.R., Hirt C.J., Andersson I.J., Renshall L.J., Baker B.C., Baker P.N., Sibley C.P., Wareing M. (2012). Enos knockout mouse as a model of fetal growth restriction with an impaired uterine artery function and placental transport phenotype. Am. J. Physiol. Regul. Integr. Comp. Physiol..

[17] Stanley J.L., Andersson I.J., Hirt C.J., Moore L., Dilworth M.R., Chade A.R., Sibley C.P., Davidge S.T., Baker P.N. (2012). Effect of the anti-oxidant tempol on fetal growth in a mouse model of fetal growth restriction. Biol. Reprod..

[18] McCarthy F.P., Kingdom J.C., Kenny L.C., Walsh S.K. (2011). Animal models of preeclampsia; uses and limitations. Placenta.

[19] Gilbert J.S., Babcock S.A., Granger J.P. (2007). Hypertension produced by reduced uterine perfusion in pregnant rats is associated with increased soluble fms-like tyrosine kinase-1 expression. Hypertension.

[20] Salas S.P., Altermatt F., Campos M., Giacaman A., Rosso P. (1995). Effects of long-term nitric oxide synthesis inhibition on plasma volume expansion and fetal growth in the pregnant rat. Hypertension.

[21] Molnar M., Suto T., Toth T., Hertelendy F. (1994). Prolonged blockade of nitric oxide synthesis in gravid rats produces sustained hypertension, proteinuria, thrombocytopenia, and intrauterine growth retardation. Am. J. Obstet. Gynecol..

[22] Yallampalli C., Garfield R.E. (1993). Inhibition of nitric oxide synthesis in rats during pregnancy produces signs similar to those of preeclampsia. Am. J. Obstet. Gynecol..

[23] Murphy S.R., LaMarca B., Cockrell K., Arany M., Granger J.P. (2012). l-arginine supplementation abolishes the blood pressure and endothelin response to chronic increases in plasma sFlt-1 in pregnant rats. Am. J. Physiol. Regul. Integr. Comp. Physiol..

[24] Murphy S.R., LaMarca B.B., Cockrell K., Granger J.P. (2010). Role of endothelin in mediating soluble fms-like tyrosine kinase 1-induced hypertension in pregnant rats. Hypertension.

[25] Maynard S.E., Min J.Y., Merchan J., Lim K.H., Li J., Mondal S., Libermann T.A., Morgan J.P., Sellke F.W., Stillman I.E. (2003). Excess placental soluble fms-like tyrosine kinase 1 (sFlt1) may contribute to endothelial dysfunction, hypertension, and proteinuria in preeclampsia. J. Clin. Investig..

[26] Kumasawa K., Ikawa M., Kidoya H., Hasuwa H., Saito-Fujita T., Morioka Y., Takakura N., Kimura T., Okabe M. (2011). Pravastatin induces placental growth factor (PGF) and ameliorates preeclampsia in a mouse model. Proc. Natl. Acad. Sci. USA.

[27] Cui Y., Wang W., Dong N., Lou J., Srinivasan D.K., Cheng W., Huang X., Liu M., Fang C., Peng J. (2012). Role of corin in trophoblast invasion and uterine spiral artery remodelling in pregnancy. Nature.

[28] Kanasaki K., Palmsten K., Sugimoto H., Ahmad S., Hamano Y., Xie L., Parry S., Augustin H.G., Gattone V.H., Folkman J. (2008). Deficiency in catechol-o-methyltransferase and 2-methoxyoestradiol is associated with pre-eclampsia. Nature.

[29] Doridot L., Passet B., Mehats C., Rigourd V., Barbaux S., Ducat A., Mondon F., Vilotte M., Castille J., Breuiller-Fouche M. (2013). Preeclampsia-like symptoms induced in mice by fetoplacental expression of stox1 are reversed by aspirin treatment. Hypertension.

[30] Davisson R.L., Hoffmann D.S., Butz G.M., Aldape G., Schlager G., Merrill D.C., Sethi S., Weiss R.M., Bates J.N. (2002). Discovery of a spontaneous genetic mouse model of preeclampsia. Hypertension.

[31] Dokras A., Hoffmann D.S., Eastvold J.S., Kienzle M.F., Gruman L.M., Kirby P.A., Weiss R.M., Davisson R.L. (2006). Severe feto-placental abnormalities precede the onset of hypertension and proteinuria in a mouse model of preeclampsia. Biol. Reprod..

[32] Kublickiene K.R., Cockell A.P., Nisell H., Poston L. (1997). Role of nitric oxide in the regulation of vascular tone in pressurized and perfused resistance myometrial arteries from term pregnant women. Am. J. Obstet. Gynecol..

[33] Poston L., McCarthy A.L., Ritter J.M. (1995). Control of vascular resistance in the maternal and feto-placental arterial beds. Pharmacol. Ther..

[34] Schiessl B., Strasburger C., Bidlingmaier M., Mylonas I., Jeschke U., Kainer F., Friese K. (2006). Plasma- and urine concentrations of nitrite/nitrate and cyclic guanosinemonophosphate in intrauterine growth restricted and preeclamptic pregnancies. Arch. Gynecol. Obstet..

[35] Krause B.J., Carrasco-Wong I., Caniuguir A., Carvajal J., Farias M., Casanello P. (2013). Endothelial enos/arginase imbalance contributes to vascular dysfunction in iugr umbilical and placental vessels. Placenta.

[36] Lassala A., Bazer F.W., Cudd T.A., Datta S., Keisler D.H., Satterfield M.C., Spencer T.E., Wu G. (2010). Parenteral administration of l-arginine prevents fetal growth restriction in undernourished ewes. J. Nutr..

[37] Vosatka R.J., Hassoun P.M., Harvey-Wilkes K.B. (1998). Dietary l-arginine prevents fetal growth restriction in rats. Am. J. Obstet. Gynecol..

[38] Satterfield M.C., Dunlap K.A., Keisler D.H., Bazer F.W., Wu G. (2013). Arginine nutrition and fetal brown adipose tissue development in nutrient-restricted sheep. Amino Acids.

[39] Rytlewski K., Olszanecki R., Korbut R., Zdebski Z. (2005). Effects of prolonged oral supplementation with l-arginine on blood pressure and nitric oxide synthesis in preeclampsia. Eur. J. Clin. Investig..

[40] Sieroszewski P., Suzin J., Karowicz-Bilinska A. (2004). Ultrasound evaluation of intrauterine growth restriction therapy by a nitric oxide donor (l-arginine). J. Matern. Fetal Neona.

[41] Xiao X.M., Li L.P. (2005). l-arginine treatment for asymmetric fetal growth restriction. Int. J. Gynaecol. Obstet..

[42] Facchinetti F., Longo M., Piccinini F., Neri I., Volpe A. (1999). l-arginine infusion reduces blood pressure in preeclamptic women through nitric oxide release. J. Soc. Gynecol. Investig..

[43] De Pace V., Chiossi G., Facchinetti F. (2007). Clinical use of nitric oxide donors and l-arginine in obstetrics. J. Matern. Fetal Neona.

[44] Villanueva-Garcia D., Mota-Rojas D., Hernandez-Gonzalez R., Sanchez-Aparicio P., Alonso-Spilsbury M., Trujillo-Ortega M.E., Necoechea R.R., Nava-Ocampo A.A. (2007). A systematic review of experimental and clinical studies of sildenafil citrate for intrauterine growth restriction and pre-term labour. J. Obstet. Gynaecol..

[45] Wareing M., Myers J.E., O’Hara M., Baker P.N. (2005). Sildenafil citrate (viagra) enhances vasodilatation in fetal growth restriction. J. Clin. Endocrinol. Metab..

[46] Stanley J.L., Andersson I.J., Poudel R., Rueda-Clausen C.F., Sibley C.P., Davidge S.T., Baker P.N. (2012). Sildenafil citrate rescues fetal growth in the catechol-O-methyl transferase knockout mouse model. Hypertension.

[47] Dilworth M.R., Andersson I., Renshall L.J., Cowley E., Baker P., Greenwood S., Sibley C.P., Wareing M. (2013). Sildenafil citrate increases fetal weight in a mouse model of fetal growth restriction with a normal vascular phenotype. PLoS ONE.

[48] Ramesar S.V., Mackraj I., Gathiram P., Moodley J. (2011). Sildenafil citrate decreases sFlt-1 and seng in pregnant l-name treated sprague-dawley rats. Eur. J. Obstet. Gynecol. Reprod. Biol..

[49] Herraiz S., Pellicer B., Serra V., Cauli O., Cortijo J., Felipo V., Pellicer A. (2012). Sildenafil citrate improves perinatal outcome in fetuses from pre-eclamptic rats. BJOG.

[50] Lacassie H.J., Germain A.M., Valdes G., Fernandez M.S., Allamand F., Lopez H. (2004). Management of eisenmenger syndrome in pregnancy with sildenafil and l-arginine. Obstet. Gynecol..

[51] Molelekwa V., Akhter P., McKenna P., Bowen M., Walsh K. (2005). Eisenmenger’s syndrome in a 27 week pregnancy—management with bosentan and sildenafil. Ir. Med. J..

[52] Samangaya R.A., Mires G., Shennan A., Skillern L., Howe D., McLeod A., Baker P.N. (2009). A randomised, double-blinded, placebo-controlled study of the phosphodiesterase type 5 inhibitor sildenafil for the treatment of preeclampsia. Hypertens. Pregnancy.

[53] Von Dadelszen P., Dwinnell S., Magee L.A., Carleton B.C., Gruslin A., Lee B., Lim K.I., Liston R.M., Miller S.P., Rurak D. (2011). Sildenafil citrate therapy for severe early-onset intrauterine growth restriction. BJOG.

[54] Ganzevoort W., Alfirevic Z., von Dadelszen P., Kenny L., Papageorghiou A., Gluud C., van Wassenaer-Leemhuis A., Mol B.W., Baker P.N. (2014). Strider: Sildenafil therapy in dismal prognosis early-onset intrauterine growth restriction—A protocol for a systematic review with individual participant data and aggregate data meta-analysis and trial sequential analysis. Syst. Rev..

[55] Myatt L. (2006). Placental adaptive responses and fetal programming. J. Physiol..

[56] Hoffmann D.S., Weydert C.J., Lazartigues E., Kutschke W.J., Kienzle M.F., Leach J.E., Sharma J.A., Sharma R.V., Davisson R.L. (2008). Chronic tempol prevents hypertension, proteinuria, and poor feto-placental outcomes in bph/5 mouse model of preeclampsia. Hypertension.

[57] Poudel R., Stanley J.L., Rueda-Clausen C.F., Andersson I.J., Sibley C.P., Davidge S.T., Baker P.N. (2013). Effects of resveratrol in pregnancy using murine models with reduced blood supply to the uterus. PLoS ONE.

[58] Roberts V.H., Pound L.D., Thorn S.R., Gillingham M.B., Thornburg K.L., Friedman J.E., Frias A.E., Grove K.L. (2014). Beneficial and cautionary outcomes of resveratrol supplementation in pregnant nonhuman primates. FASEB J..

[59] Thakor A.S., Herrera E.A., Seron-Ferre M., Giussani D.A. (2010). Melatonin and vitamin c increase umbilical blood flow via nitric oxide-dependent mechanisms. J. Pineal Res..

[60] Niu Y., Herrera E.A., Evans R.D., Giussani D.A. (2013). Antioxidant treatment improves neonatal survival and prevents impaired cardiac function at adulthood following neonatal glucocorticoid therapy. J. Physiol..

[61] Chappell L.C., Seed P.T., Briley A.L., Kelly F.J., Lee R., Hunt B.J., Parmar K., Bewley S.J., Shennan A.H., Steer P.J. (1999). Effect of antioxidants on the occurrence of pre-eclampsia in women at increased risk: A randomised trial. Lancet.

[62] Chappell L.C., Seed P.T., Kelly F.J., Briley A., Hunt B.J., Charnock-Jones D.S., Mallet A., Poston L. (2002). Vitamin c and e supplementation in women at risk of preeclampsia is associated with changes in indices of oxidative stress and placental function. Am. J. Obstet. Gynecol..

[63] Poston L., Briley A.L., Seed P.T., Kelly F.J., Shennan A.H. (2006). Vitamin c and vitamin e in pregnant women at risk for pre-eclampsia (vip trial): Randomised placebo-controlled trial. Lancet.

[64] Reiter R.J., Tan D.X. (2003). Melatonin: A novel protective agent against oxidative injury of the ischemic/reperfused heart. Cardiovasc. Res..

[65] Miller S.L., Yawno T., Alers N.O., Castillo-Melendez M., Supramaniam V.G., VanZyl N., Sabaretnam T., Loose J.M., Drummond G.R., Walker D.W. (2014). Antenatal antioxidant treatment with melatonin to decrease newborn neurodevelopmental deficits and brain injury caused by fetal growth restriction. J. Pineal Res..

[66] Richter H.G., Hansell J.A., Raut S., Giussani D.A. (2009). Melatonin improves placental efficiency and birth weight and increases the placental expression of antioxidant enzymes in undernourished pregnancy. J. Pineal Res..

[67] Hobson S.R., Lim R., Gardiner E.E., Alers N.O., Wallace E.M. (2013). Phase i pilot clinical trial of antenatal maternally administered melatonin to decrease the level of oxidative stress in human pregnancies affected by pre-eclampsia (pampr): Study protocol. BMJ Open.

[68] Alers N.O., Jenkin G., Miller S.L., Wallace E.M. (2013). Antenatal melatonin as an antioxidant in human pregnancies complicated by fetal growth restriction—A phase i pilot clinical trial: Study protocol. BMJ Open.

[69] Hypponen E., Cavadino A., Williams D., Fraser A., Vereczkey A., Fraser W.D., Banhidy F., Lawlor D., Czeizel A.E. (2013). Vitamin d and pre-eclampsia: Original data, systematic review and meta-analysis. Ann. Nutr. Metab..

[70] Chen Y.H., Fu L., Hao J.H., Yu Z., Zhu P., Wang H., Xu Y.Y., Zhang C., Tao F.B., Xu D.X. (2015). Maternal vitamin d deficiency during pregnancy elevates the risks of small for gestational age and low birth weight infants in chinese population. J. Clin. Endocrinol. Metab..

[71] Harvey N.C., Holroyd C., Ntani G., Javaid K., Cooper P., Moon R., Cole Z., Tinati T., Godfrey K., Dennison E. (2014). Vitamin d supplementation in pregnancy: A systematic review. Health Technol. Assess..

[72] Henderson J.T., Whitlock E.P., O’Conner E., Senger C.A., Thompson J.H., Rowland M.G. (2014). Low-Dose Aspirin for the Prevention of Morbidity and Mortality from Preeclampsia: A Systematic Evidence Review for the U.S. Preventive Services Task Force.

[73] Ludman A., Venugopal V., Yellon D.M., Hausenloy D.J. (2009). Statins and cardioprotection—More than just lipid lowering?. Pharmacol. Ther..

[74] Costantine M.M., Tamayo E., Lu F., Bytautiene E., Longo M., Hankins G.D., Saade G.R. (2010). Using pravastatin to improve the vascular reactivity in a mouse model of soluble fms-like tyrosine kinase-1-induced preeclampsia. Obstet. Gynecol..

[75] Saad A.F., Kechichian T., Yin H., Sbrana E., Longo M., Wen M., Tamayo E., Hankins G.D., Saade G.R., Costantine M.M. (2014). Effects of pravastatin on angiogenic and placental hypoxic imbalance in a mouse model of preeclampsia. Reprod. Sci..

[76] Bauer A.J., Banek C.T., Needham K., Gillham H., Capoccia S., Regal J.F., Gilbert J.S. (2013). Pravastatin attenuates hypertension, oxidative stress, and angiogenic imbalance in rat model of placental ischemia-induced hypertension. Hypertension.

[77] Forbes K., Hurst L.M., Aplin J.D., Westwood M., Gibson J.M. (2008). Statins are detrimental to human placental development and function; use of statins during early pregnancy is inadvisable. J. Cell. Mol. Med..

[78] Forbes K., Shah V.K., Siddals K., Gibson J.M., Aplin J.D., Westwood M. (2015). Statins inhibit insulin-like growth factor action in first trimester placenta by altering insulin-like growth factor 1 receptor glycosylation. Mol. Hum. Reprod..

[79] Kapil V., Weitzberg E., Lundberg J.O., Ahluwalia A. (2014). Clinical evidence demonstrating the utility of inorganic nitrate in cardiovascular health. Nitric Oxide.

[80] Cottrell E., Garrod A., Wareing M., Dilworth M., Finn-Sell S., Greenwood S., Baker P., Sibley C. (2014). Supplementation with inorganic nitrate during pregnancy improves maternal uterine artery function and placental efficiency in mice. Placenta.

[81] Cottrell E., Garrod A., Finn-Sell S., Wareing M., Cowley E., Greenwood S., Baker P.N., Sibley C. (2015). Maternal supplementation with dietary inorganic nitrate improves uteroplacental vascular function in enos(−/−) mice. Reprod. Sci..

[82] Matsui D. (2012). Adherence with drug therapy in pregnancy. Obstet. Gynecol. Int..

[83] David A.L., Torondel B., Zachary I., Wigley V., Abi-Nader K., Mehta V., Buckley S.M., Cook T., Boyd M., Rodeck C.H. (2008). Local delivery of vegf adenovirus to the uterine artery increases vasorelaxation and uterine blood flow in the pregnant sheep. Gene Ther..

[84] Mehta V., Abi-Nader K.N., Shangaris P., Shaw S.W., Filippi E., Benjamin E., Boyd M., Peebles D.M., Martin J., Zachary I. (2014). Local over-expression of VEGF-ddeltandeltac in the uterine arteries of pregnant sheep results in long-term changes in uterine artery contractility and angiogenesis. PLoS ONE.

[85] Carr D.J., Wallace J.M., Aitken R.P., Milne J.S., Mehta V., Martin J.F., Zachary I.C., Peebles D.M., David A.L. (2014). Uteroplacental adenovirus vascular endothelial growth factor gene therapy increases fetal growth velocity in growth-restricted sheep pregnancies. Hum. Gene Ther..

[86] Harris L., Kotamraju R., Teesalu T., Ruoslahti E. (2012). Identification of novel placental homing peptides. Placenta.

[87] King A., Tirelli N., Aplin J., Harris L. (2014). Targeted delivery of insulin-like growth factor-II to the placenta using homing peptide-decorated liposomes increases placental weight. Placenta.

[88] Cureton N., Cellesi F., Aplin J., Harris L. (2013). Homing peptide-mediated targeting of liposomes to term villous explants: Novel nanocarriers for targeted drug delivery. Placenta.

